# Cell competition within breast tumor microenvironment: emerging role of cancer stem cells and immune cells in tumor progression and therapeutic implications

**DOI:** 10.3389/fonc.2026.1861308

**Published:** 2026-07-22

**Authors:** Esha Pal, Sovan Chakraborty, Aishwarya Guha

**Affiliations:** 1 Department of Genetic Toxicology, Krish Biotech Research Private Limited, Kalyani, India; 2 Department of Biotechnology, National Institute of Technology, Warangal, India; 3 Department of Biotechnology, Swami Vivekananda University, Kolkata, India

**Keywords:** breast cancer stem cells, cell competition, immune cells, metabolic reprogramming, therapeutic targeting, tumor microenvironment, tumor-associated macrophages, Warburg effect

## Abstract

Breast cancer remains the most prevalent malignancy among women worldwide, with approximately 2.3 million new cases diagnosed annually, accounting for nearly 12% of all cancer diagnosis globally. Increasing evidence highlights the tumor microenvironment (TME) as a dynamic and interactive ecosystem that governs tumor behaviour. This review consolidates emerging evidence that positions cell competition as a critical regulator of clonal dynamics within the breast cancer TME. We examine how breast cancer stem cells (BCSCs), defined by CD44^+^/CD24^-^ phenotype and elevated ALDH activity, acquire enhanced fitness through MYC amplification and dysregulation of Hippo/YAP, Wnt/β-catenin and Notch signaling pathways. In parallel, we analyze the role of immune cell populations including tumor-associated macrophages, cytotoxic T lymphocytes, natural killer cells and myeloid-derived suppressor cells in shaping competitive interactions through resource limitation and immuno-suppressive signaling. We further explore metabolic competition, highlighting the Warburg effect, reverse Warburg effect and lactate-mediated immuno-suppression as central regulators of cellular fitness, alongside contributions from cancer-associated fibroblasts, extracellular matrix remodeling and exosome-mediated communication. The novelty of this study lies in integrating cellular, metabolic and stromal dimensions of competition into a unified framework and extending the concept beyond tumor cells to include immune and non-cellular components. Cumulatively, this study identifies competitive cellular fitness as a central and targetable driver of therapeutic resistance and tumor recurrence in breast cancer.

## Graphical Abstract

**Figure:**
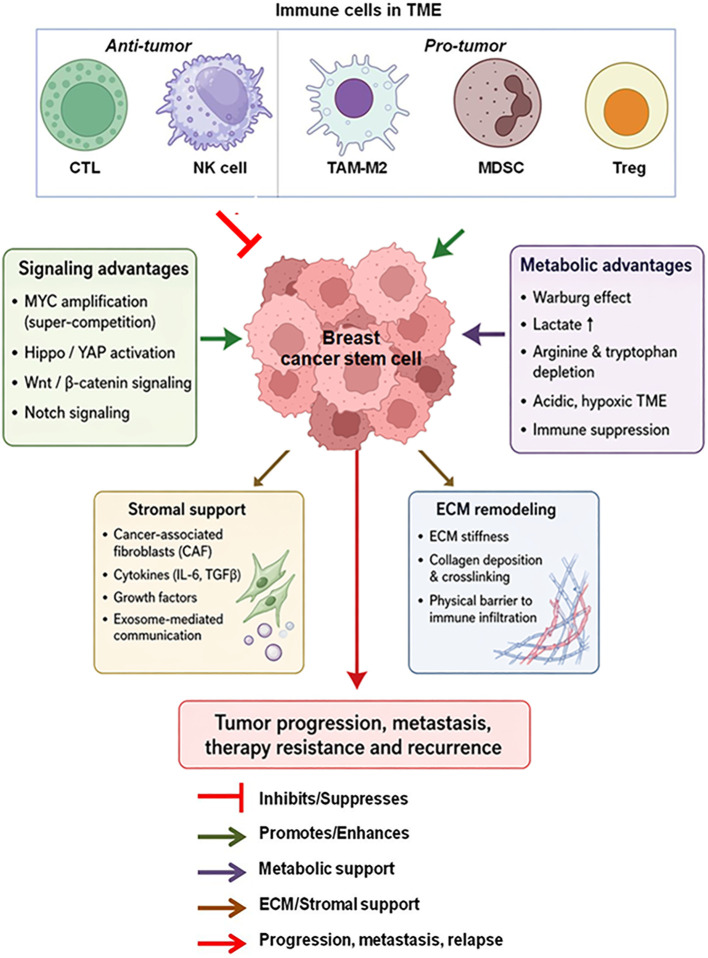
Breast cancer stem cells acquire enhanced competitive fitness through activation of key signaling pathways, including MYC, Hippo/YAP, Wnt/β-catenin and Notch, which promote self-renewal, proliferation and elimination of less fit neighboring cells. Concurrently, metabolic reprogramming via the Warburg effect and lactate accumulation generates an acidic and nutrient-deprived microenvironment that suppresses anti-tumor immune cell function. Breast cancer stem cells further reshape the TME through interactions with stromal components, including cancer-associated fibroblasts (CAFs), extracellular matrix (ECM) remodeling and exosome-mediated communication, which collectively support tumor growth and create physical and biochemical barriers to immune infiltration. Immune cell populations exhibit opposing competitive roles: cytotoxic T lymphocytes (CTLs) and natural killer (NK) cells mediate anti-tumor responses, whereas tumor-associated macrophages (TAMs), myeloid-derived suppressor cells (MDSCs) and regulatory T cells (Tregs) contribute to immunosuppression.

## 1 Introduction

Breast cancer is the most frequently diagnosed cancer in women globally, representing an alarming public health burden. An estimated 2.3 million incident cases and approximately 670,000 deaths has been reported in 2022 according to GLOBOCAN data (1). Despite remarkable advances in early detection, molecular subtyping and targeted therapy, metastatic breast cancer continues to carry a 5-years survival rate of approximately 32%. This underscores the persistent need for novel conceptual frameworks to guide therapeutic development (2). A major impediment to treatment efficacy is the complexity of the breast tumor, which must be understood as a dynamic ecosystem encompassing stromal cells, immune populations, the extracellular matrix (ECM) and a rich soluble milieu of cytokines, chemokines, growth factors, and metabolites collectively constituting the tumor microenvironment (TME) (3).

The concept of cell competition, first described in *Drosophila melanogaster* by Morata and Ripoll in 1975, refers to the elimination of viable but less fit cells (‘losers’) by their more fit neighbours (‘winners’). This maintains tissue homeostasis and optimizes organ composition during normal development (4). Winners instruct losers to undergo apoptosis and itself expand clonally to fill the vacant niche. Seminal work identified that cells expressing elevated levels of the proto-oncogene MYC behave as ‘supercompetitors,’ actively outcompeting and eliminating wild-type neighbouring cells (5). This mechanism is evolutionarily conserved from *Drosophila* to mammals and has been documented in mammalian tissues and increasingly recognized as a major force operating within human tumors (6).

In breast cancer, the TME is especially amenable to competitive dynamics. Breast tumors are characterized by marked intratumoral heterogeneity, with subpopulations of cells exhibiting vastly different proliferative capacities, metabolic activities and sensitivities to therapeutic agents (7). At the apex of this heterogeneous hierarchy resides breast cancer stem cells (BCSCs), defined by their CD44^+^/CD24^−^/^low^ phenotype or elevated aldehyde dehydrogenase (ALDH) activity, which constitute a small but disproportionately influential fraction of the tumor mass (8). BCSCs demonstrate exquisite self-renewal capacity, resistance to conventional cytotoxic therapies and remarkable plasticity in inter-converting between stem and non-stem states in response to micro-environmental cues (8, 9). Immune cells particularly tumor-associated macrophages (TAMs), CD8^+^ cytotoxic T lymphocytes (CTLs), NK cells and myeloid-derived suppressor cells (MDSCs) are abundant in the breast TME and engage in intricate competitive interactions that are increasingly recognized as determinants of tumor fate (10).

This review encompasses the emerging body of evidence on cell competition within the breast TME, focusing on the contributions of CSCs and immune cells in this process. We address the molecular signaling mechanisms including Hippo/YAP, Wnt/β-catenin, Notch and MYC pathways that mediate competitive fitness. We elucidate metabolic competition as a central mechanism by which cancer cells and immune cells contest niche resources, with specific attention to the Warburg effect, lactate-mediated immuno-suppression and amino acid depletion. Finally, we outline therapeutic strategies that leverage emerging insights into cell competition to devise more effective treatment combinations for breast cancer.

## 2 Cell competition in normal and malignant tissues

### 2.1 Historical origins and mechanistic principles

The original description of cell competition in *Drosophila* Minute mutants revealed that haploid-insufficient ribosomal protein mutations caused cells to be outcompeted and eliminated by adjacent wild-type cells, even though the mutant cells were intrinsically viable (4, 5). This observation established several defining principles of cell competition: (i) the outcome of competition is contextual and dependent on relative rather than absolute fitness (ii) elimination of loser cells involves induction of apoptosis via cell-non-autonomous signals (iii) winner cells undergo compensatory proliferation and (iv) the overall tissue maintains homeostatic size (11). Subsequent identification that MYC overexpression transforms cells into supercompetitors which not only survive but actively induce apoptosis in neighbouring wild-type cells revealed a critical intersection between canonical oncogene biology and competitive interactions (5).

In mammals, cell competition has been demonstrated during early embryogenesis, in adult homeostatic tissues and critically, within tumors (12). Mechanistic studies have identified several molecular readouts of cellular fitness. The Flower protein family, first characterized in *Drosophila*, encodes isoforms whose differential expression ‘Flower-Win’ versus ‘Flower-Lose’ provides a molecular code that allows cells to compare fitness levels with their neighbours (13). Mammalian homologs of Flower have been identified and their expression correlates with competitive outcomes in experimental models. The Hippo pathway effectors YAP and TAZ, which integrate mechanical, biochemical and proliferative cues, have been identified as major mammalian mediators of cell competition (14). Epithelial cell delamination (EDAC), in which normal epithelial cells sense and eliminate oncogene expressing neighbours through Filamin and Vimentin-dependent mechanisms, represents another conserved competition mechanism of direct relevance to early tumorigenesis (15).

### 2.2 Cell competition in cancer: the tumor as a competitive arena

Within tumors, cell competition functions on multiple levels simultaneously. Inter-clonal competition bestows distinct mutational lineages against one another in contests for physical space, oxygen, nutrients and signaling factor access. Cancer cells also compete with adjacent normal stromal cells, with accumulated evidence suggesting that cancer cells frequently adopt supercompetitor behaviours that eliminate surrounding fibroblasts and normal epithelial cells. A landmark study demonstrated that loss of APC or upregulation of MYC, in tumor cells generates supercompetitors that actively outcompete wild-type neighbours (16). Additionally, cancer-microenvironment competition is mediated through cytokine signaling, metabolic exchange, exosomal communication and mechanical force transmission.

In the breast cancer TME, competitive dynamics are particularly complex given the cellular density and functional heterogeneity of the microenvironment. Single-cell RNA sequencing (scRNA-seq) studies have revealed coexisting subpopulations with markedly different transcriptional states, suggesting ongoing competitive selection (17). Spatial transcriptomics has resolved the distinct geographic architecture of breast tumors, demonstrating that mesenchymal like and stem-like cancer cells are preferentially enriched at the invasive leading edge and tumor-stroma interface-the precise microenvironmental niches where active, heterotypic cellular interactions drive progression (18). Evolutionary game-theoretic models applied to tumor heterogeneity data demonstrate that frequency-dependent competitive and cooperative interactions among diverse subclones account for a significant fraction of the variance in clonal composition, extending beyond what would be expected from neutral drift or autonomous proliferation alone (19).

## 3 Cancer stem cells as supercompetitors in the breast cancer TME

### 3.1 Defining the breast cancer stem cell phenotype

BCSCs were first prospectively identified by Al-Hajj et al. in 2003, who demonstrated that as few as 100 CD44^+^/CD24^−^/ESA^+^ cells could efficiently regenerate human breast tumors in NOD/SCID mice, whereas ten thousand CD44^−^/CD24^+^ cells failed to do so (20). Subsequent work defined ALDH activity particularly the ALDH1A1 isoform as an alternative or complementary BCSC marker, with ALDH^+^ cells demonstrating enhanced tumorigenicity and metastatic capacity (8). Importantly, BCSCs do not constitute a static population; rather, plasticity between stem and non-stem phenotypes is driven by microenvironmental signals including hypoxia, TGF-β, inflammatory cytokines and physical stiffness of the ECM (21). This plasticity is central to competitive dynamics, as non-stem cancer cells can acquire stem traits under selective pressure, thereby generating new competitive subpopulations within the tumor.

**Figure 1:**
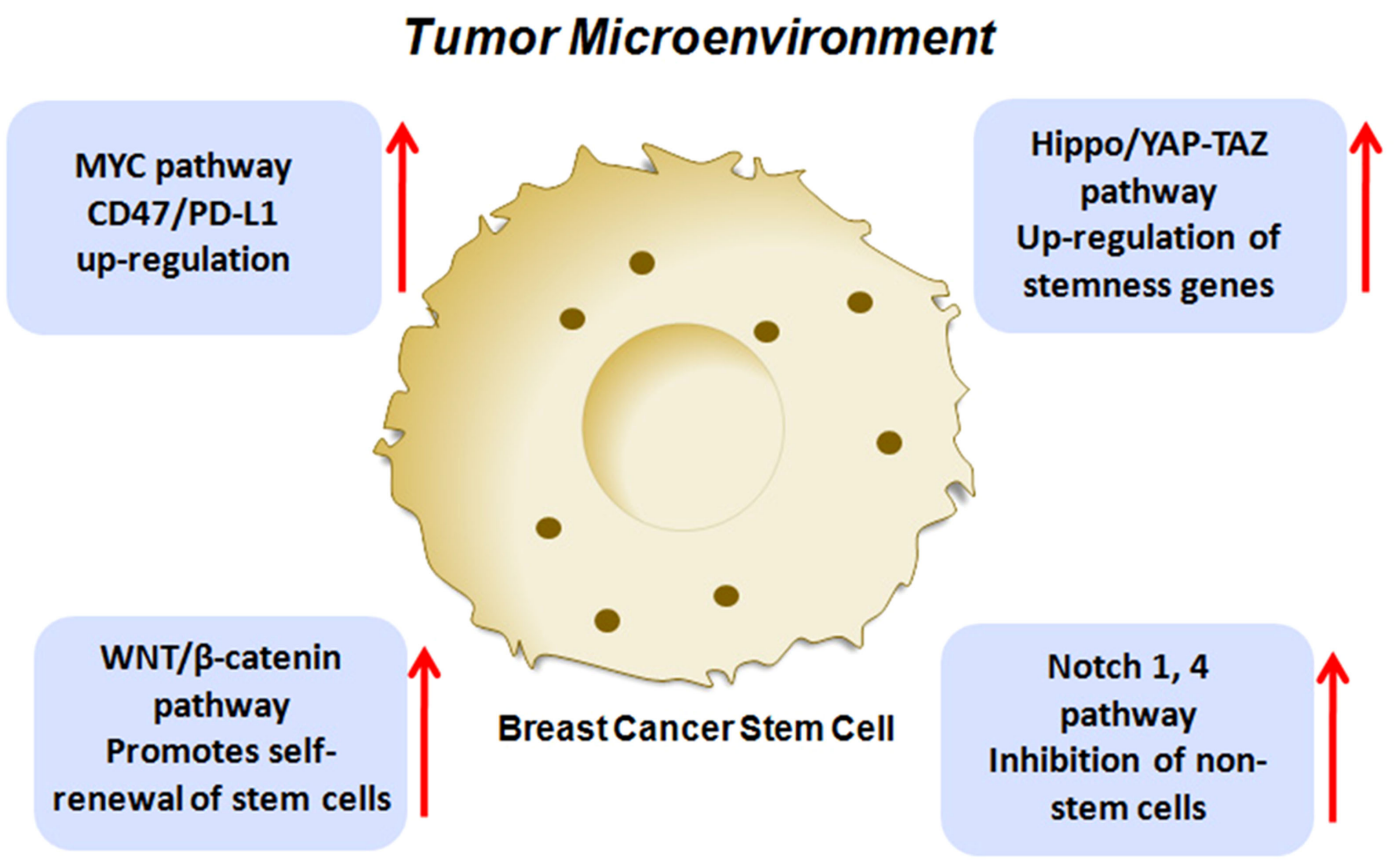
BCSC signaling pathways as competitive fitness mediators: schematic representation of molecular pathways showing the four key signaling axes that makes BCSCs super-competitors within the TME. This includes MYC mediated apoptosis induction in neighbouring cells along with CD47/PD-L1 upregulation, Hippo/YAP-TAZ signalling facilitating mechano-sensing and upregulation of stemness regulating genes, Wnt/β-catenin promotes self-renewal of BCSCs along with Frizzled-mediated niche signal sequestration, Notch1/4 promotes lateral inhibition of non-stem cancer cells.

At the molecular level, BCSCs are characterized by constitutive activation of multiple stemness-promoting signaling networks. Wnt/β-catenin signaling promotes self-renewal and maintains an undifferentiated transcriptional state (22) (**Figure 1**). Notch signaling, particularly through Notch1 and Notch4 receptors and their Jagged/Delta-like ligands, governs BCSC fate decisions and maintains a proliferative advantage in competitive contexts (**Figure 1**). Hedgehog signaling, acting through Gli transcription factors, sustains BCSC populations especially in the triple-negative breast cancer (TNBC) subtype (23). The Hippo pathway effector YAP/TAZ has emerged as a particularly important mediator of BCSC competitive fitness, as its nuclear accumulation drives stem gene expression, promotes resistance to anoikis and mediates mechanosensing of ECM stiffness (24).

### 3.2 MYC-mediated supercompetition and its role in BCSC dominance

MYC amplification or over-expression is observed in approximately 15-25% of all breast cancers and is strongly associated with TNBC and basal-like subtypes (25). At the mechanistic level, elevated MYC confers super-competitor status upon cancer cells. MYC-high cells actively induce apoptosis in MYC-low neighbours via caspase-3-dependent mechanisms, concurrently accelerating their own proliferation to fill the vacated niche. Di Giacomo et al. demonstrated stereotypical patterns of MYC-mediated cell competition in human cancer specimens, with apoptotic cells concentrated at the tumor-stroma interface (26) (**Figure 1**). Further, a significant correlation between MYC protein level in the tumor and cell death amount in the stromal compartment, irrespective of tumor type or stage has been reported (26). These observations suggest that MYC-driven cell competition is an active process occurring continuously at the tumor-stroma boundary in breast and other cancers.

In breast cancer specifically, MYC amplification is frequently co-acquired with other oncogenic events that enhance competitive fitness. MYC overexpression has been shown to upregulate CD47-the ‘do not eat me’ signal and PD-L1, thereby simultaneously enabling evasion of phagocytosis and adaptive immune surveillance (**Figure 1**) (27). This dual competitive advantage enables MYC-amplified BCSCs to both outcompete neighbouring stromal cells and evade immune cell elimination.

### 3.3 Hippo/YAP pathway as a mediator of BCSC competitive fitness

The Hippo signaling pathway, canonically involving the MST1/2-LATS1/2 kinase cascade that phosphorylates and inactivates YAP/TAZ transcriptional coactivators, has emerged as a critical regulator of cell competition fitness in mammalian tissues (14). In breast cancer, dysregulation of Hippo signaling leads to nuclear accumulation of YAP/TAZ, which drives transcription of genes promoting proliferation, survival and stemness, including CTGF, CYR61 and BIRC5 (28). Importantly, YAP/TAZ activity is mechanosensitive responding to ECM stiffness and cell density positioning the Hippo pathway as a sensor of the physical competitive landscape within the TME (**Figure 1**). In breast tumors, stiff and crosslinked ECM, a hallmark of the desmoplastic response, activates YAP/TAZ in BCSCs and tumor cells, conferring mechanical competitive advantage over more compliant stromal cells. Studies have demonstrated that YAP activation in peritumoral epithelial cells can paradoxically suppress adjacent tumor cell outgrowth through EDAC-like mechanisms, suggesting that the direction of Hippo-mediated competition depends critically on cellular context (14).

YAP/TAZ functionally intersects with other BCSC stemness pathways, creating an integrated competitive signaling network. YAP cooperates with Wnt/β-catenin to co-regulate stem gene expression and YAP stabilization reciprocally amplifies Wnt signaling by preventing cytoplasmic sequestration of β-catenin (29, 30). Interaction with Notch signaling occurs through TAZ stabilization by the Notch intracellular domain, establishing a self-reinforcing loop that maintains BCSC competitive dominance in a densely signaled microenvironment. Furthermore, in TNBC, RAD18-mediated YAP activation promotes TAM polarization towards the pro-tumorigenic M2 phenotype through TGF-β, creating a positive feedback loop in which BCSCs not only compete directly against other cell types but also remodel the immune components of the TME to suppress anti-tumor competition (31).

### 3.4 Wnt/β-Catenin and notch pathways in BCSC niche competition

Niche competition in the breast cancer TME involves not only direct cell-cell competitive interactions but also competition for access to niche-supporting signals, including Wnt ligands, Notch ligands and growth factors. BCSCs express high levels of Frizzled receptors and LRP5/6 co-receptors, enabling efficient capture of Wnt ligands secreted by stromal cells including TAMs and CAFs (22). This competitive sequestration of niche signals depletes the local Wnt availability for non-stem cancer cells and normal mammary epithelial cells, reinforcing the hierarchical dominance of BCSCs. In xenograft models of breast cancer, experimental reduction of BCSC Wnt signaling disrupted niche competition and promoted differentiation of the tumor bulk thereby reducing tumorigenicity (32).

Notch signaling in breast cancer operates through a particularly elegant competitive mechanism. Notch activation requires direct cell-cell contact between ligand-expressing and receptor-expressing cells, creating short-range competitive gradients within the tumor mass. BCSCs expressing high levels of Notch1 preferentially receive survival and self-renewal signals from adjacent Jagged-1-expressing TAMs and CAFs, while simultaneously engaging in lateral inhibition suppressing Notch activation in neighbouring cancer cells through feedback mechanisms. This short-range competitive circuit contributes to the maintenance of a minority BCSC fraction within the tumor hierarchy (**Figure 1**). Therapeutic disruption of this Notch-mediated niche competition using gamma-secretase inhibitors has demonstrated efficacy in reducing BCSC frequency and tumor initiation capacity in preclinical breast cancer models (9).

## 4 Immune cell competition within the breast cancer TME

### 4.1 Tumor-associated macrophages: dual competitors and collaborators

TAMs represent one of the most abundant immune cell type in the breast cancer TME, comprising 30-50% of the infiltrated immune cells in many breast tumors. TAMs are functionally heterogeneous, existing on a spectrum between classically activated (M1-like) anti-tumor phenotypes and alternatively activated (M2-like) pro-tumor phenotypes. However, this binary classification incompletely justifies their actual functional diversity as revealed by single-cell analysis (33, 34). M1-like TAMs engage in anti-competitive behaviours against cancer cells through direct cytotoxicity, phagocytosis and secretion of pro-inflammatory cytokines including TNF-α, IL-12 and CXCL9/10/11 that recruit additional anti-tumor effector cells. M2-like TAMs, may conversely support BCSC maintenance through secretion of IL-6, IL-10, TGF-β and EGF, establishing a cooperative rather than competitive relationship with the tumor cell hierarchy (35).

A pivotal 2024 study in Nature Cancer demonstrated that TAMs in the fibrotic breast cancer TME undergo metabolic reprogramming driven by TGF-β, initiating a collagen biosynthesis program that creates a metabolically hostile milieu for CD8^+^ T cells through arginine consumption and ornithine secretion (36)This TAM-mediated metabolic competition directly inhibits cytotoxic T cell function and survival, illustrating a sophisticated competitive mechanism by which M2-like TAMs eliminate competing anti-tumor immune populations. The study demonstrated this arginine-ornithine axis as a critical competitive mediator in female breast cancer, providing a mechanistic basis for the inverse correlation between fibrosis, TAM infiltration and T cell functionality observed clinically. These findings underscore the importance of considering TAMs not merely as regulators of cancer cell behaviour, but as active participants in multi-cellular competitive dynamics that determine immune landscape composition.

**Figure 2:**
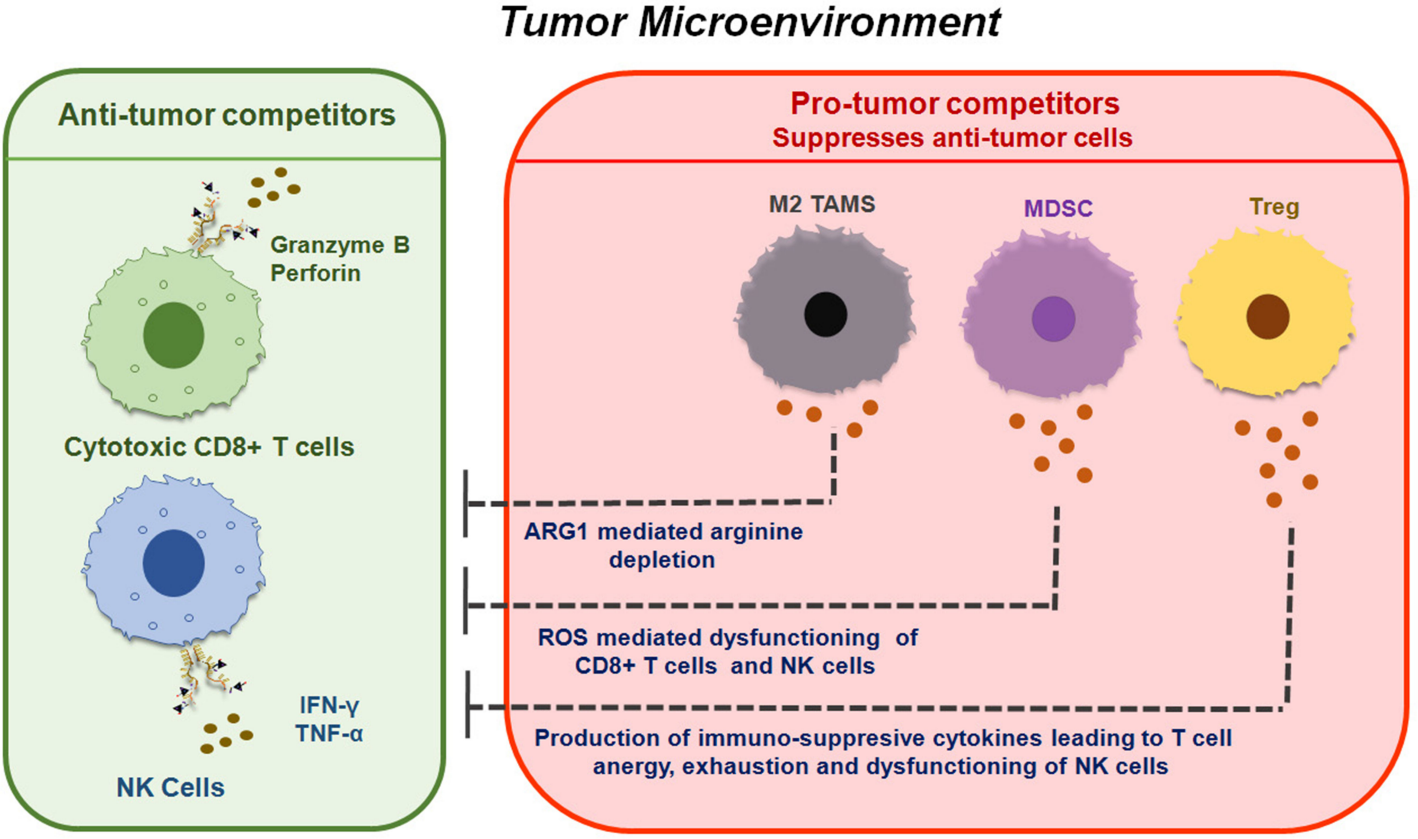
Immune cell competitive dynamics within the breast cancer tumor microenvironment. Schematic representation of the opposing competitive forces exerted by anti-tumor and pro-tumor immune cell populations within the breast tumor microenvironment (TME). Anti-tumor competitors, including CD8^+^ cytotoxic T lymphocytes (CTLs) and natural killer (NK) cells, engage breast cancer cells and BCSCs through cytotoxic mechanisms mediated by perforin, granzymes and IFN-γ secretion. These anti-tumor populations are progressively suppressed by pro-tumor immunosuppressive cells comprising M2-polarized tumor-associated macrophages (TAMs), myeloid-derived suppressor cells (MDSCs) and regulatory T cells (Tregs). M2 TAMs suppress CTL and NK cell function through arginase-1 (ARG1)-mediated arginine depletion. MDSCs impair effector immune cell function via reactive oxygen species (ROS) production. Tregs suppress CTL proliferation and NK cell functioning by producing immunosuppressive cytokines.

The M1/M2 TAM balance constitutes a competitive equilibrium within the TME, dynamically influenced by cancer cell-derived signals, metabolic conditions and spatial organization. A high M1/M2 ratio is associated with improved survival across multiple solid tumor types, reflecting the suppression of pro-tumorigenic competitive interactions by anti-tumor TAMs. Breast cancer cells subvert this equilibrium by secreting CSF-1, CCL-2, CSF-5 that polarize TAMs toward M2 phenotypes, thereby reshaping the competitive landscape in their favour (37). High-resolution spatial transcriptomics of human breast tumors has revealed that M2-like TAMs are preferentially enriched in hypoxic and stromal regions which precisely are the zones where BCSCs reside (**Figure 2**).

### 4.2 Cytotoxic T lymphocytes and regulatory T cells: competitive dynamics

Tumor-infiltrating lymphocytes (TILs), particularly CD8^+^ CTLs, represent the primary adaptive immune competitors against breast cancer cells. High TIL density in TNBC is associated with improved pathological complete response rates and better survival outcomes, reflecting successful immune competition against tumor cells (38). However, the TME in established breast tumors extensively suppresses CTL competitive capacity through multiple mechanisms. PD-L1 expression on cancer cells and M2 TAMs engages with the PD-1 inhibitory receptor on CTLs, attenuating TCR signaling and metabolic activity, impairing the competitive fitness of CTLs in a glucose-depleted microenvironment. BCSCs are particularly adept at PD-L1-mediated immune evasion. BCSCs have been shown to express higher PD-L1 levels than their non-stem counterparts, creating a competitive shield around the stem cell niche (39).

Regulatory T cells (Tregs) constitute a critical opposing competitive force against CTLs within the breast cancer TME. TAM-secreted chemokines including CCL22 and CCL17 recruit CCR4^+^ Tregs to the TME (40), where they suppress CTL activity through IL-10, TGF-β and IL-35 secretion, consumption of the T cell growth factor IL-2 and contact-dependent CTLA-4/CD80–86 competitive ligand binding. The ratio of CTL to Treg infiltration has been proposed as a prognostic biomarker in breast cancer, with high CTL/Treg ratios associated with improved outcomes (41). From a competitive perspective, the CTL/Treg balance represents a quantifiable outcome of ongoing immune cell competition for survival signals and niche space within the TME. Several studies have estimated the competitive exclusion coefficients between CTL and Treg populations, proving that Treg competitive advantage increases markedly under hypoxic and lactate-rich conditions characteristic of metabolically active tumors (42) (**Figure 2**).

### 4.3 Natural killer cells: innate competitive sentinels

NK cells represent innate immune competitors that do not require prior sensitization to recognize and eliminate cancer cells. NK cell activation is governed by a balance of activating and inhibitory signals. Cancer cells expressing stress ligands (NKG2D ligands) without inhibitory HLA class I molecules are preferentially eliminated, while cancer cells that downregulate activating ligands or overexpress HLA class I evade NK cell competition. BCSCs have been shown to be particularly resistant to NK cell-mediated elimination through multiple competitive evasion strategies. CD44 over-expression on BCSCs inhibits NK cell-activating pathways, while TGF-β secreted by both BCSCs and M2 TAMs directly suppresses NK cell cytotoxicity and promotes exhaustion. In addition, BCSCs express high levels of CD47 which engages SIRPα on NK cells and macrophages, reducing phagocytic competition against the stem cell population (43, 44).

The spatial organization of NK cells within breast tumors reflects the outcome of competitive displacement by immunosuppressive forces. In TNBC, NK cells are frequently excluded from the tumor core and restricted to peritumoral regions, consistent with active competitive displacement mediated by CXCR4-CXCL12 chemokine gradients established by CAFs and BCSCs that direct NK cells away from the tumor parenchyma. Restoration of NK cell competitive access to the tumor core through CXCR4 blockade or CXCL12 neutralization represents a promising therapeutic strategy to reinstate innate anti-tumor competition. Recent studies have also demonstrated that lactate-a major metabolic waste product of glycolytic BCSCs directly suppresses NK cell competitive fitness by impairing cytolytic granule release, establishing a mechanistic link between metabolic competition and innate immune competition in breast cancer (45) (**Figure 2**).

### 4.4 Myeloid derived suppressor cells: competitive immune-suppression

MDSCs constitute a heterogeneous population of immature myeloid cells that accumulate in breast cancer and potently suppress anti-tumor immune competition (46). MDSCs exert competitive suppression against CTLs and NK cells through multiple metabolic and contact-dependent mechanisms, including production of reactive oxygen species (ROS), arginase-1-mediated arginine depletion, iNOS-derived nitric oxide and IL-10 secretion. MDSCs also directly compete with CTLs for amino acid substrates in the TME, creating a nutritional disadvantage for effector immune cells. The recruitment of MDSCs to the breast cancer TME is driven by cancer cell-derived cytokines like VEGF, IL-6, G-CSF and GM-CSF (46, 47). Importantly, BCSCs are potent inducers of MDSC recruitment and activation, suggesting that the competitive dominance of BCSCs is partially achieved through MDSC-mediated suppression of competing immune effectors. Recent studies have revealed functionally distinct MDSC subsets in breast cancer, with poly-morphonuclear MDSCs (PMN-MDSCs) preferentially accumulating in TNBC and correlating with poor responses to immunotherapy (46, 48) (**Figure 2**).

## 5 Metabolic competition in the breast cancer TME

### 5.1 The Warburg effect as a competitive metabolic strategy

Cancer cells in the breast cancer TME compete aggressively for limited metabolic substrates, particularly glucose, glutamine, oxygen and amino acids. Warburg effect, the preferential conversion of glucose to lactate even in the presence of adequate oxygen dramatically increases the glucose consumption rate of cancer cells relative to normal cells, depleting shared glucose pools and creating metabolic disadvantage for competing immune and stromal populations. In TNBC, the Warburg effect is particularly pronounced, driven by HIF-1α-mediated transcriptional upregulation of glycolytic enzymes (GLUT1, HK2, PFKFB3, LDHA) and monocarboxylate transporters (MCT4) (49). Lactate, the primary metabolic product of the Warburg effect in breast cancer cells, profoundly reshapes competitive dynamics within the TME through multiple mechanisms. High extracellular lactate concentrations (up to 40 mmol/L in some solid tumors) acidify the TME to pH 6-7, impair CTL cytotoxic function, suppress NK cell activation and promote M2 TAM polarization, collectively suppressing immune cell competition against cancer (50). Lactate also serves as a signaling molecule. It activates the lactate receptor GPR81 on cancer cells and immune cells, triggering downstream pathways that promote cancer cell survival while impairing immune cell function. In breast cancer, lactate-mediated suppression of immune competition has been directly linked to therapy resistance, with high serum lactate dehydrogenase (LDH) being associated with resistance to anti-PD-1 therapy.

Beyond its immunosuppressive functions, lactate is increasingly recognized as an active signaling metabolite capable of influencing CSC biology. Emerging evidence suggests that elevated extracellular lactate can promote stemness-associated phenotypes through metabolic reprogramming and activation of signaling pathways linked to cellular plasticity, self-renewal and therapeutic resistance. Furthermore, lactate may serve as an alternative metabolic substrate for stem-like tumor cell populations, supporting oxidative metabolism and enhancing their adaptability within nutrient-restricted microenvironments. In breast cancer, these effects may reinforce the competitive fitness of BCSCs by facilitating their survival under metabolically challenging conditions while simultaneously suppressing anti-tumor immune responses. Consequently, lactate may function not only as a mediator of metabolic competition but also as a contributor to the maintenance of stem-like cellular states that drive tumor progression and treatment resistance. Nevertheless, the precise contribution of lactate-driven stemness to competitive interactions between BCSCs, non-stem cancer cells and immune populations remains incompletely understood and warrants further investigation (50, 51).

In addition, ischemic and hypoxic regions within tumors further intensify metabolic competition by limiting oxygen and nutrient availability while promoting adaptive cellular responses. These conditions can enhance the survival of stem-like cancer cell populations through HIF-1α-mediated metabolic reprogramming and increased cellular plasticity, potentially conferring a competitive advantage within the tumor hierarchy. However, the extent to which ischemia-driven stemness directly influences competitive interactions among BCSCs, non-stem cancer cells and immune populations remains to be fully elucidated (21).

### 5.2 The reverse Warburg effect and metabolic symbiosis

The reverse Warburg effect, introduced by Pavlides and colleagues, describes a metabolic symbiosis in which cancer cells induce aerobic glycolysis in adjacent CAFs through oxidative stress signaling and then import CAF-derived lactate and pyruvate via MCT1 for mitochondrial oxidative phosphorylation (OXPHOS) (51)This metabolic division of labor enables cancer cells to sustain high OXPHOS activity essential for the biosynthetic demands of proliferation while externalizing the metabolic burden of glycolysis to the stroma. In breast cancer, the reverse Warburg effect has been particularly well characterized in estrogen receptor-positive and HER2-positive tumors, where CAF-derived lactate fuels aggressive, OXPHOS-dependent cancer cell populations (52). BCSCs preferentially exploit this metabolic symbiosis: Accumulating evidence reveals that lactate mitochondrial oxidation is a primary driver of stemness potential in metastatic breast cancer. Consequently, ALDH1^+^ BCSCs exhibit significantly higher OXPHOS activity than their non-stem counterparts, establishing a strict dependency on MCT1-mediated lactate import to siphon metabolic fuel directly from glycolytic CAFs and surrounding non-stem cancer cells (53).

The metabolic competition for lactate between BCSCs and non-stem cancer cells creates an intra-tumoral competitive gradient in which cells best positioned to exploit available metabolic substrates acquire proliferative and survival advantages. This competitive metabolic geography overlaps with the BCSC niche at the perivascular region, where oxygen tension is adequate for OXPHOS and where CAF-derived lactate is locally available (54). Single-cell metabolomics studies integrating spatial information have begun to map these metabolic competition landscapes within individual tumors, revealing heterogeneous metabolic states coexisting within millimeters of each other and identifying spatially defined competitive interfaces between OXPHOS-dominant and glycolysis-dominant subpopulations (55).

### 5.3 Amino acid competition and immuno-metabolic crosstalk

Beyond glucose competition, amino acid competition represents an increasingly recognized arena of cell competition in the breast cancer TME. Arginine, tryptophan and glutamine are particularly critical competitive substrates for immune cells. Arginine is essential for CTL and NK cell proliferation, cytokine production and cytotoxic function. Its depletion by arginase-1 (ARG1) expressed by M2 TAMs and MDSCs creates a competitive metabolic barrier for anti-tumor immune cells. In the fibrotic breast cancer TME, as demonstrated by the 2024 Nature Cancer study, TAMs undergo TGF-β-induced metabolic reprogramming that increases ARG1 activity, consuming environmental arginine and generating ornithine as a byproduct that further suppresses T cell function, establishing an elegant competitive metabolic circuit entirely mediated by TAMs (36). Tryptophan catabolism through the IDO1/TDO2-kynurenine axis depletes tryptophan from the TME, impairing CTL and NK cell function while generating kynurenine, which activates the aryl hydrocarbon receptor (AhR) in Tregs and promotes their differentiation and suppressive function (56). In breast cancer, IDO1 expression is upregulated in cancer cells, BCSCs and TAMs in response to IFN-γ secreted by competing CTLs. This creates an elegant competitive feedback loop in which the signals of CTL activation trigger tryptophan depletion that competitively suppresses CTL survival and function (57). Glutamine competition adds a further layer of complexity. Both cancer cells (for purine synthesis, hexosamine pathway and redox balance) and T cells (for TCA cycle anaplerosis and proliferation) depend heavily on glutamine, creating a contest for this essential substrate within the TME (58).

## 6 Competition between cellular and non-cellular TME components

### 6.1 Cancer-associated fibroblasts as competitive arbiters

CAFs are the most abundant stromal cell type in the breast cancer TME, comprising up to 80% of the stromal compartment in desmoplastic breast tumors. CAFs are functionally heterogeneous, with single-cell transcriptomic studies identifying distinct subtypes including myofibroblastic CAFs (myCAFs), inflammatory CAFs (iCAFs), antigen-presenting CAFs (apCAFs), vascular CAFs (vCAFs) and matrix CAFs (mCAFs), each exhibiting different competitive behaviours within the TME (59). myCAFs, characterized by αSMA expression and contractile morphology, produce dense collagen matrices that create physical barriers impeding immune cell infiltration and representing a form of non-cellular competitive exclusion of anti-tumor immunity (60). iCAFs secrete pro-tumorigenic cytokines including IL-6, IL-8, CXCL1 and LIF that promote BCSC self-renewal and survival, constituting a competitive cooperation with the cancer stem cell fraction.

The competitive interactions between CAFs and BCSCs are bidirectional and mutually reinforcing (61, 62). Cancer cells particularly BCSCs activate quiescent normal fibroblasts into CAFs through TGF-β, PDGF and FGF secretion, creating a reinforcing loop in which BCSCs generate their own niche-supporting stromal partners. Conversely, CAFs maintain CSC stemness through secretion of paracrine factors including HGF, NF-kB activation, Wnt ligands and stromal-derived factor-1 (SDF-1/CXCL12), which activate competitive signaling cascades within BCSCs (63, 64). Exosomal communication between CAFs and BCSCs enables horizontal transfer of oncogenic mRNAs, miRNAs and proteins that enhance BCSC competitive fitness, including resistance to chemotherapy and anoikis (65). From a competitive perspective, CAFs function as infrastructure-building allies of BCSCs, creating a permissive niche environment that systematically disadvantages competing immune and stromal populations.

### 6.2 Extracellular matrix remodeling as a competitive non-cellular force

The ECM within the breast cancer TME is not merely a passive component but an active competitive modulator that confers mechanical advantages to cells capable of exploiting its physical properties (66–68). ECM stiffness generated by collagen crosslinking through LOX and LOXL enzymes secreted by CAFs activates YAP/TAZ in adjacent cancer cells and BCSCs via integrin-mediated mechano-transduction, promoting proliferation, survival and competitive dominance. High mammographic density demonstrating ECM stiffness is one of the strongest risk factors for breast cancer development and aggressive phenotype, consistent with a role for ECM-mediated competitive advantage in tumor initiation and progression. The transition from ductal carcinoma *in situ* (DCIS) to invasive breast cancer has been associated with progressive changes in ECM structure and composition that correlate with the emergence of CAF populations competitively remodeling the stroma (69, 70).

ECM remodeling also influences immune cell competition by creating physical barriers that restrict CTL and NK cell penetration into the tumor parenchyma-a major mechanism of resistance to immunotherapy in breast cancer (71). Collagen density at the tumor-stroma interface has been shown to physically impede CTL infiltration and therapeutic targeting of collagen remodeling using LOX inhibitors has improved CTL access to tumor cells and enhanced response to anti-PD-1 therapy in preclinical breast cancer models. The ECM also sequesters competitive cytokines and growth factors including TGF-β, FGF and VEGF, controlling their bioavailability and thereby shaping the competitive signaling landscape. Heparan sulfate proteoglycans within the ECM act as molecular storage depots that regulate competitive access to Wnt ligands which are critical niche signals for stemness maintenance (72). This creates a competition for morphogen binding to non-cellular matrix components.

### 6.3 Exosomal and soluble factor competition

Exosomes are extracellular vesicles of endosomal origin which constitute a critical medium for competitive communication within the breast cancer TME. BCSC-derived exosomes contain functional Wnt ligands, epigenetic regulators (including EZH2 and DNMT1 loaded mRNAs), and competitive signaling molecules that can reprogram recipient cells, either converting non-stem cancer cells toward a BCSC phenotype (competitive cooperation) or suppressing immune cell function (competitive suppression). CAF-derived exosomes reciprocally enhance BCSC stemness and chemotherapy resistance by delivering oncogenic miRNAs and metabolic enzymes that shift BCSC metabolism toward OXPHOS-dependent competitive fitness (73). In addition to exosomes, soluble factors including cytokines, chemokines and metabolites establish competitive gradients within the TME. Competition for cytokine signals particularly IL-2, a limiting T cell survival factor consumed by both CTLs and Tregs creates a quantifiable competitive disadvantage for CTLs in established tumors with high Treg infiltration (74). VEGF, secreted in high amounts by hypoxic BCSCs, competitively recruits endothelial cells to form tumor-associated vasculature, shunting angiogenic resources away from normal tissue homeostasis and creating a BCSC-preferential vascular niche (75). PD-L1 expressing exosomes released by breast cancer cells and TAMs engage PD-1 on CTLs at a distance from the primary tumor, suppressing immune surveillance of circulating tumor cells and early metastatic seeds, extending the competitive reach of the immunosuppressive TME into systemic circulation (76).

## 7 Probabilistic and quantitative dimensions of cell competition

Quantitative modelling of cell competition in the breast cancer TME has evolved from simple proliferative models to sophisticated stochastic and agent-based frameworks that incorporate spatial heterogeneity, metabolic constraints and evolutionary dynamics. The Moran process traditionally a model of neutral evolution in finite populations has been successfully extended to cancer frameworks to evaluate non-neutral clonal dynamics by incorporating asymmetric fitness variables. When evaluating the stem cell niche under favourable microenvironmental conditions, these stochastic boundaries allow for the modeling of competitive advantages between cancer stem cells (CSCs) and non-CSCs (77, 78).

Evolutionary game theory approaches have effectively modeled the competitive relationships between cooperating and defecting cancer cell strategies within the tumor microenvironment (19). Public goods games in which cooperator cells incur a fitness cost to produce paracrine growth factors that benefit neighbouring free-riders predict dynamic evolutionary cycling that maintains phenotypic tumor heterogeneity. Complementing these theoretical frameworks, large-scale genomic analyses of cohorts such as the 560 breast cancer whole-genome dataset (79) and the METABRIC cohort confirm deep subclonal heterogeneity. Deconvolution of this sequencing data demonstrates that subclonal architectures are strongly shaped by active competitive selection at both the gene and subclonal level, rather than a simple, neutral accumulation of random mutations (80).

## 8 Therapeutic implications of cell competition in breast cancer

### 8.1 Immune checkpoint inhibitors: restoring immune competitive balance

Immune checkpoint inhibitors (ICIs) targeting the PD-1/PD-L1 axis represent the most clinically advanced strategy to restore immune competitive balance in breast cancer (81). In TNBC-the subtype with the highest tumor mutational burden, TIL density and PD-L1 expression, pembrolizumab, an anti-PD-1 antibody, has received FDA approval in combination with chemotherapy for both metastatic PD-L1-positive disease (based on the KEYNOTE-522 and KEYNOTE-355 trials) and for early-stage high-risk disease in the neoadjuvant setting (82, 83). By blocking the PD-1/PD-L1 competitive suppression axis, ICIs restore CTL competitive fitness within the TME, enabling immune effectors to successfully compete against and eliminate cancer cell.

Despite these advances, ICI response rates in breast cancer remains limited to approximately 20-40% of TNBC patients and 9-11% significant increase in pathologic complete response rates particularly in early stage HR^+^/HER2^-^ and HER2^+^ patients (84). From a cell competition perspective, this limited efficacy reflects incomplete restoration of immune competitive balance, as multiple redundant competitive suppression mechanisms remain active even after PD-1/PD-L1 blockade. TAM-mediated arginine depletion, Treg recruitment via CCL22, MDSC-driven ROS production and physical ECM-mediated immune exclusion continue to impair CTL competitive fitness independently of PD-1 signaling (85). This mechanistic insight argues for combination strategies that simultaneously target multiple competitive suppression axis. This is currently being tested in numerous clinical trials by combining ICIs with CSF-1R inhibitors (TAM reprogramming), Treg-depleting therapies (anti-CCR8, anti-CTLA-4) and IDO inhibitors (86).

### 8.2 Targeting cancer stem cell competitive pathways

Therapeutic targeting of BCSC competitive fitness pathways represents a critical complementary strategy to immunotherapy. Wnt pathway inhibitors including porcupine inhibitors (LGK-974, WNT-974), Frizzled receptor antagonists (OMP-54F28) and β-catenin/TCF interaction disruptors have demonstrated efficacy in reducing BCSC frequency and sensitizing breast tumors to conventional therapy in preclinical models (87). Several Wnt pathway inhibitors are currently in Phase I/II clinical trials for advanced solid tumors including breast cancer. Notch inhibition through gamma-secretase inhibitors or anti-Notch receptor antibodies reduces BCSC competitive self-renewal capacity and enhances the sensitivity of BCSCs to competing immune attack (88). Targeting the YAP/TAZ competitive fitness axis in breast cancer has been explored through verteporfin (a YAP inhibitor), TEAD transcription factor inhibitors and upstream mechano-sensing pathway modulators, with promising preclinical results that are being translated into clinical investigation (24).

ALDH inhibition strategies targeting the BCSC competitive marker aldehyde dehydrogenase have been explored using disulfiram and ALDH-specific small molecule inhibitors, with dual effects of reducing BCSC stemness and enhancing sensitivity to both chemotherapy and immunotherapy (89). The CD44^+^/CD24^-^ surface phenotype of BCSCs has been exploited for antibody-drug conjugate (ADC) delivery, enabling targeted cytotoxic molecule delivery to the competitive stem cell fraction. An important emerging strategy involves targeting MYC-mediated super-competition directly, using MYC inhibitors such as BET bromodomain inhibitors that suppress MYC transcription, thereby abrogating the competitive advantage conferred by MYC amplification on BCSCs (90, 91). Combining MYC inhibition with immune checkpoint blockade has the potential to simultaneously reduce the competitive fitness of BCSCs and restore immune competitive surveillance.

### 8.3 Metabolic reprogramming as competitive therapy

Given the central role of metabolic competition in the breast cancer TME, therapies that target the metabolic competitive advantage of cancer cells represent a promising dimension of competitive cancer therapy. Glycolysis inhibitors targeting GLUT1 (BAY-876), HK2 (2-deoxyglucose, 2-DG), PFKFB3 and LDHA seek to reduce cancer cell glucose consumption and lactate production, thereby alleviating the metabolic competitive pressure on immune cells (92). While selective toxicity against cancer cells is a challenge due to overlapping metabolic dependencies between cancer cells and rapidly dividing immune cells careful dosing and scheduling strategies may exploit the greater metabolic plasticity of immune cells relative to highly glycolytic cancer cells. MCT1 and MCT4 inhibitors targeting lactate transport have demonstrated promise in reducing lactate-mediated immunosuppression in breast cancer models (93).

Diet and systemic metabolic interventions represent another level at which competitive metabolic dynamics in the breast cancer TME can be modified. Ketogenic diet and intermittent fasting reduce systemic glucose availability, reducing the competitive glycolytic advantage of breast cancer cells while potentially enhancing immune cell metabolic fitness through ketone body utilization. Dietary restriction of specific amino acids including methionine and glutamine has shown selective anti-tumor competitive effects in preclinical models, impairing cancer cell competitive fitness more than immune cell function (94). IDO1 inhibitors including epacadostat have been tested in combination with pembrolizumab in early breast cancer trials, targeting tryptophan competitive depletion as an immunosuppressive mechanism (94). Although the phase III ECHO-301/KEYNOTE-252 trial of epacadostat plus pembrolizumab failed in melanoma, subsequent analysis suggested that insufficient pathway blockade and absence of biomarker-guided selection may have contributed. Therefore, improved IDO inhibitor design and stratification using tryptophan-kynurenine pathway biomarkers could remain relevant in breast cancer (95).

### 8.4 CAF-directed and ECM-modifying strategies

Targeting CAFs and the ECM competitive infrastructure they create represents a complementary therapeutic approach to disrupting pro-tumor competitive dynamics in breast cancer. FAP-targeted CAR-T cell therapy eliminates immunosuppressive FAP+ CAFs, reducing their competitive support of the BCSC niche and restoring immune infiltration (96). Anti-fibrotic agents including pirfenidone and nintedanib reduce ECM stiffness and CAF activation, indirectly improving immune competitive access to tumor parenchyma. LOX inhibitor simtuzumab, targeting the primary collagen cross-linking enzyme, has been evaluated in clinical trials for solid tumors, with the rationale of reducing ECM-mediated immune exclusion (97). CXCL12/CXCR4 axis blockade using plerixafor (AMD3100) in combination with ICIs is currently being investigated in clinical trials (98).

Exosome biogenesis inhibition is an emerging strategy to disrupt tumor microenvironment communication in breast cancer. Key regulators such as Rab27a/b and neutral sphingomyelinase-2 (nSMase2) control multivesicular body trafficking, docking, and ceramide-dependent exosome formation; their inhibition reduces exosome secretion and may impair tumor progression and stromal signaling (99). Engineered therapeutic exosomes loaded with anti-tumor nucleic acids, including miRNA inhibitors, tumor-suppressive miRNAs/mRNAs, and other gene-therapy constructs, are being investigated as delivery vehicles capable of modulating tumor microenvironment interactions and enhancing anti-tumor responses (100). The table below (**Table 1**) summarizes the key competitive mechanisms and the therapeutic strategies to target them.

**Table 1:** Therapeutic strategies against the various competitive mechanisms.

Competitive mechanism	Therapeutic strategy
PD-1/PD-L1 immune-suppression	Pembrolizumab (ICI)
Wnt BCSC niche competition	LGK-974, OMP-54F28
YAP/TAZ competitive fitness	Verteporfin, TEAD inhibitors
MYC supercompetition	BET inhibitors, 10058-F4
Warburg metabolic advantage	GLUT1/HK2/LDHA inhibitors
Arginine/tryptophan depletion	IDO1 inhibitors (epacadostat)
CAF competitive support	FAP-CAR-T, pirfenidone
ECM immune exclusion	LOX inhibitors, CXCR4 blockade
Exosomal competitive communication	nSMase2 inhibitors

## 9 Discussion

This review has incorporated the emerging evidence that cell competition is a fundamental organizational principle of the breast cancer TME, operating through multiple complementary mechanisms that collectively determine tumor fate. The conceptual framework of cell competition in which outcomes are governed by relative rather than absolute fitness provides compelling evidences to understand the complex, context-dependent behaviour of BCSCs, immune cells, CAFs and the ECM within breast tumors. BCSCs are not passive beneficiaries of a permissive TME but active architects of competitive dominance. An important but often underappreciated aspect of BCSC biology is the dynamic plasticity that enables bidirectional transitions between stem and non-stem cellular states. Rather than representing a fixed population, BCSCs exist in a state of continuous equilibrium with differentiated tumor cells, allowing adaptation to changing microenvironmental conditions and therapeutic pressures. These transitions are governed by several molecular switches, including epithelial-to-mesenchymal transition (EMT), mesenchymal-to-epithelial transition (MET) and stemness-associated signaling pathways such as Wnt/β-catenin, Notch, Hippo/YAP-TAZ and TGF-β. Activation of transcription factors including OCT4, SOX2, NANOG, KLF4 and c-MYC promotes maintenance of the stem-like phenotype, whereas attenuation of these programs facilitates differentiation toward non-stem cellular states. Initiation of Wnt/β-catenin signaling promotes nuclear accumulation of β-catenin, leading to the transcription of stemness-associated genes including OCT4, SOX2 and NANOG, thereby favouring maintenance or reacquisition of the stem-cell phenotype. Notch signaling on the other hand regulates cell-fate decisions through ligand-dependent cleavage of Notch receptors and nuclear translocation of the Notch intracellular domain (NICD), which promotes self-renewal while suppressing differentiation programs. Similarly, TGF-β signaling induces EMT, a process strongly associated with the acquisition of stem-like properties, increased plasticity and therapeutic resistance. In contrast, reversal of EMT through MET is frequently associated with partial loss of stemness and cellular differentiation. Hippo pathway dysregulation, resulting in nuclear localization of YAP/TAZ, enhances expression of genes involved in self-renewal, survival and cellular plasticity, thereby facilitating dedifferentiation of non-stem cancer cells into stem-like states. Collectively, these pathways act as dynamic molecular switches that determine cellular identity and govern the equilibrium between stem and non-stem populations within the breast TME.

These molecular switches are tightly regulated by signals originating from distinct components of the TME. CAFs represent a major source of Wnt ligands, CXCL12, HGF and IL-6, which activate β-catenin, STAT3 and NF-κB signaling pathways to promote self-renewal and dedifferentiation. Similarly, M2-polarized TAMs secrete TGF-β, EGF and Jagged1, thereby inducing EMT and activating Notch signaling in neighbouring tumor cells, which facilitates acquisition and maintenance of stem-like characteristics. Hypoxic niches within the tumor stabilize HIF-1α and HIF-2α, resulting in enhanced expression of stemness-associated genes including OCT4, SOX2 and NANOG. In parallel, ECM remodeling and increased matrix stiffness activate integrin-dependent mechanotransduction pathways that promote YAP/TAZ nuclear localization and stemness-associated transcriptional programs. Collectively, these findings indicate that the TME not only provides a supportive niche for pre-existing BCSCs but also actively orchestrates the molecular events that drive transitions between stem and non-stem states, thereby contributing to tumor heterogeneity, metastatic progression and therapeutic resistance.

Through MYC amplification, Hippo/YAP activation, Wnt and Notch niche competition, metabolic engineering of the lactate rich TME and strategic deployment of immune evasion mechanisms, BCSCs systematically outcompete both non-stem cancer cell populations and anti-tumor immune populations. The plasticity of the BCSC phenotype enabling reversible interconversion between stem and non-stem states adds an additional competitive dimension, allowing the stem cell fraction to dynamically adjust its size in response to competitive pressures from therapy or immunity. An important consideration when interpreting the role of BCSCs within the TME is the marked heterogeneity among breast cancer molecular subtypes. Previous studies have demonstrated that TNBC harbors the highest frequency of BCSCs, followed by HER2^+^, luminal B and luminal A tumors, suggesting that stemness-associated pathways may contribute differentially to disease progression across subtypes. Emerging evidence further indicates that the immuno-modulatory functions of BCSCs may also exhibit subtype-specific differences. Recent findings demonstrated that BCSC-mediated CD8^+^ T-cell exhaustion is particularly prominent in aggressive breast cancer subtypes and is regulated through activation of the NOTCH1/RBPJ/PD-L1 signaling axis. These observations suggest that both the abundance and functional consequences of BCSCs are influenced by molecular subtype and should be considered when developing stemness-targeted therapeutic strategies.

Further, immune cells are not merely bystanders within competitive tumor dynamics but active participants in multi-cellular competition for space, signals and metabolic substrates. The progressive competitive displacement of CTLs and NK cells by M2 TAMs, MDSCs and Tregs mediated through metabolic depletion, signal competition and physical exclusion represents a competitive immune selection process that is as important to tumor progression as clonal selection of cancer cells themselves.

Additionally, metabolic competition deserves recognition as a fundamental competitive mechanism in breast cancer that bridges cell-intrinsic properties with TME-level dynamics. The Warburg effect, long conceptualized as a cell-autonomous metabolic adaptation can be re-conceptualized as an aggressive competitive strategy that depletes shared glucose pools, creates a hostile lactate-acidic TME for competing immune cells and establishes the reverse Warburg metabolic symbiosis with CAFs. This metabolic competition framework suggests that the success of immunotherapy in any individual tumor will depend not only on the inherent antigenicity of cancer cells or the expression of immune checkpoints, but on the metabolic competitive balance within the specific TME-a balance that varies considerably across breast cancer subtypes and individual tumors. The observation that CAF-mediated arginine competition directly suppresses T cell function in fibrotic breast cancer provides a striking example of how metabolic competition operates through non-cancer cell intermediaries.

There are important limitations to the current state of knowledge in this field. The vast majority of mechanistic studies on cell competition have been conducted in *Drosophila* or simplified cell culture systems and direct functional evidence for all the competitive mechanisms discussed herein in intact human breast tumors remains incomplete. Spatial transcriptomics and single-cell sequencing technologies are beginning to fill this gap by providing *in-situ* resolution of competitive cell state dynamics, but causal inference from such correlation-based approaches requires careful interpretation. The probabilistic and quantitative modeling frameworks discussed represent theoretical frameworks that require prospective validation in clinical datasets with well-annotated spatial TME data. Additionally, inter-individual variability in the competitive dynamics of breast cancer arising from differences in host immunity, tumor mutational burden, molecular subtype and microbiome composition challenges the generalizability of specific competitive mechanisms identified in model systems.

Thus, the integration of cell competition concepts into breast cancer research opens several exciting avenues. The development of competitive fitness biomarkers, relative MYC activity, YAP/TAZ nuclear localization, metabolic substrate consumption rates and immune cell competitive ratios within individual tumors could provide predictive information about likely competitive outcomes and optimal therapeutic interventions. The emerging field of ‘competitive therapy,’ which explicitly designs treatment combinations to shift the competitive balance within the TME rather than simply maximizing cytotoxicity against cancer cells, offers a conceptually distinct approach to overcoming therapeutic resistance. Combination therapy that simultaneously reduce BCSC competitive fitness (through Wnt, Notch, MYC or YAP/TAZ inhibition) restore immune competitive balance (through checkpoint inhibition, metabolic reprogramming and CAF modulation) and disrupt non-cellular competitive infrastructure (through ECM softening and exosome pathway targeting) may achieve synergistic effects that individually targeted approaches fail to provide.

## 10 Conclusion and future direction

Cell competition is an evolutionarily ancient biological mechanism that has been co-opted by breast cancer to drive clonal evolution, BCSC maintenance, immune evasion and therapeutic resistance. Within the breast cancer TME, cancer stem cells exploit MYC amplification, Hippo/YAP pathway dysregulation, Wnt and Notch signaling and metabolic Warburg strategy to achieve super-competitor status that enables their selective survival and expansion within a diverse cellular ecosystem. Immune cell populations including TAMs, CTLs, NK cells, Tregs, and MDSCs engage in intricate competitive dynamics for niche resources, with the progressive competitive displacement of anti-tumor immune populations by pro-tumor immunosuppressive cells representing a defining feature of breast cancer immune evasion. CAFs and the ECM provide the non-cellular competitive infrastructure that supports BCSC dominance and immune exclusion. Probabilistic and spatial mathematical models are beginning to quantify these competitive dynamics and predict therapeutic outcomes.

Translating the cell competition framework into clinical practice requires the development of novel combination therapeutic strategies that simultaneously target multiple levels of competitive regulation within the breast cancer TME. Immune checkpoint inhibitors that restore CTL competitive balance, combined with BCSC-targeting agents that reduce cancer stem cell competitive fitness, metabolic reprogramming strategies that relieve the immunosuppressive competitive metabolic environment and CAF/ECM-modifying agents that dismantle the competitive infrastructure of the desmoplastic stroma, represent a rational combinatorial framework derived from competitive principles. As spatial multi-omics technologies provide increasingly granular maps of competitive landscapes within individual breast tumors, precision competitive medicine matching therapeutic combinations to the specific competitive configuration of each patient TME may emerge as a transformative approach to treating this heterogeneous and lethal malignancy.

## Glossary

ALDH: Aldehyde dehydrogenase
ARG1: Arginase 1
BCSCs: Breast cancer stem cells
CAFs: Cancer-associated fibroblasts
CCR4: C-C chemokine receptor type 4
CSCs: Cancer stem cells
CTLs: Cytotoxic T lymphocytes
CXCL12: C-X-C motif chemokine ligand 12
CXCR4: C-X-C chemokine receptor type 4
ECM: Extracellular matrix
EDAC: Epithelial defense against cancer
EMT: Epithelial to mesenchymal transition
EZH2: Enhancer of zeste homolog 2
FGF: Fibroblast growth factor
G-CSF: Granulocyte colony-stimulating factor
GM-CSF: Granulocyte-macrophage colony-stimulating factor
HIF-1α: Hypoxia-inducible factor 1-alpha
HK2: Hexokinase 2
HLA: Human leukocyte antigen
IDO1: Indoleamine 2,3-dioxygenase 1
IFN-γ: Interferon gamma
IL: Interleukin
LDH: Lactate dehydrogenase
LDHA: Lactate dehydrogenase A
LIF: Leukemia inhibitory factor
LOX: Lysyl oxidase
MCT1/4: Monocarboxylate transporter 1/4
MDSCs: Myeloid-derived suppressor cells
MET: Mesenchymal to epithelial transition
MYC: MYC proto-oncogene
NK cells: Natural killer cells
NKG2D: Natural killer group 2D receptor
OXPHOS: Oxidative phosphorylation
PD-1: Programmed cell death protein 1
PD-L1: Programmed death-ligand 1
PFKFB3-6: Phosphofructo-2-kinase/fructose-2,6-bisphosphatase 3
ROS: Reactive oxygen species
SDF-1: Stromal cell-derived factor 1
STAT3: Signal transducer and activator of transcription 3
TAMs: Tumor-associated macrophages
TCA cycle: Tricarboxylic acid cycle
TGF-β: Transforming growth factor beta
TILs: Tumor-infiltrating lymphocytes
TME: Tumor microenvironment
TNBC: Triple-negative breast cancer
Tregs: Regulatory T cells
VEGF: Vascular endothelial growth factor
YAP: Yes-associated protein.
